# Utility of Point-of-Care Ultrasound During Prone Positioning Cardiopulmonary Resuscitation

**DOI:** 10.1155/2024/9256556

**Published:** 2024-09-14

**Authors:** Haris Patail, Tinatin Saralidze, Gabriel Hernandez Romero, Hassan Patail

**Affiliations:** ^1^ Department of Internal Medicine University of Connecticut, 263 Farmington Ave, Farmington, Connecticut 06030, USA; ^2^ Department of Internal Medicine Jacobi Medical Center, Bronx, New York, USA; ^3^ Department of Pulmonary Critical Care Medicine Jacobi Medical Center, Bronx, New York, USA

**Keywords:** ACLS, CPR, POCUS, prone position

## Abstract

This report describes a 34-year-old male admitted to the medical intensive care unit (ICU) who sustained cardiac arrest while in prone positioning. Prone position CPR was initiated, and the utilization of point-of-care ultrasound (POCUS) during CPR was necessary to assess compression quality. Specifically, the popliteal was observed using POCUS to gauge the adequacy of compressions and subsequent perfusion during prone position CPR. This approach provides insight into assessing the effectiveness of chest compressions in a challenging prone position, potentially improving outcomes in similar cases. Further research and application of POCUS in this context may enhance the quality of CPR and patient care during cardiac arrest events in prone positioning.

## 1. Introduction

Prone position CPR is a difficult technique rarely used; however, it can provide lifesaving measures and can be imperative, especially in the critical care setting. As the technique is difficult in itself, assessing for adequate compressions is extremely difficult due to inability to assess cardiac pulsations on ultrasound. The utilization of point-of-care ultrasound (POCUS) with direct visualization of other arterial vessels may provide a new ability to determine adequate CPR.

## 2. Case Presentation

A 34 year-old-male with a past medical history of diabetes mellitus, ethanol (EtOH) use disorder, prior admission 1 year prior with myocarditis and diffuse alveolar hemorrhage (DAH) treated with steroids, and left lower extremity deep venous thrombosis (DVT) presented to the emergency department with 6 h of chest pain, abdominal pain, nausea, and vomiting. On arrival, he was diaphoretic and expectorating pink and frothy sputum. His presenting vitals showed a blood pressure of 99/45 mmHg, heart rate of 125 beats per min, and peripheral oxygen saturation (SpO_2_) of 85% on room air. Physical exam was remarkable for bilateral rhonchi, abdominal guarding, and cold extremities. Early in the hospital course, his condition rapidly deteriorated and he sustained worsening acute hypoxemic respiratory failure requiring endotracheal intubation. Laboratory studies were remarkable for mild leukocytosis (WBC 11,260 × 10^9^ L), acute kidney injury (Cr 1.4 mg/dL), high-anion gap metabolic acidosis (anion gap 28.3 and bicarbonate 17.7 mmol/L), and lactic acidosis (lactate 8.5 mmol/L). Venous blood gas showed pH 7.13 and pCO_2_ 76. Cardiac enzymes, coagulation parameters, electrolytes, and lipase were within normal limits. Computerized tomography angiogram of the chest and abdomen showed diffuse pulmonary ground glass opacities with superimposed dependent dense consolidations and interlobular septal thickening. Initial POCUS showed diffuse pulmonary B-line patterns with hepatization of lung parenchyma and decreased left ventricular function without pericardial effusion. His SpO_2_ was persistently between 85% and 88% despite providing 100% fraction of inspired oxygen (FiO_2_), thus requiring proning position ventilation with improvement of SpO_2_ to 94%. Due to presumed septic shock, the patient was initiated on vasopressors including norepinephrine, phenylephrine, and vasopressin infusions. Despite aggressive vasopressor support, he continued to have refractory shock with mean arterial pressures of <65 mmHg. Given an ongoing respiratory failure, shock state, and profound acidosis, the patient sustained cardiac arrest with pulseless electrical activity.

ACLS protocol was initiated while the patient was prone to minimize the potential interruption in no/low flow states. In order to ensure adequate compressions while in the prone position, POCUS was used to identify the popliteal artery. During compressions, the popliteal was accurately visualized for pulsations to ensure adequate CPR quality. ROSC was achieved and the patient was then carefully repositioned to supine position. Another brief cardiac arrest ensued while supine; however, ROSC was quickly achieved again. Total downtime, including both incidents, amounted to 20 min. Post ROSC, pink and frothy sputum was visualized in the endotracheal tube; therefore, inhaled tranexamic acid was administered in addition to 1 g of solumedrol intravenously for the treatment of presumed DAH. POCUS and chest x-ray were obtained which ruled out pneumothorax. Surrogate mixed venous saturation (SvO_2_) of 73% was obtained from the left intrajugular vein central catheter, indicating likely distributive shock with worsening lactic acidosis (pH of 7.11 and lactate of 12.5 mmol/L). Vasopressors were continued with the addition of a bicarbonate infusion which began to improve the pH to 7.26. The PaO_2_/FiO_2_ (PF) ratio remained low (68) despite once again reproning the patient.

Given objective evidence of acute respiratory distress syndrome (ARDS) in the setting of suspected DAH, the patient was placed on veno-venous (VV) extracorporeal membrane oxygenation (ECMO). Over the first 24 h, he was weaned off from vasopressors and was subsequently decannulated from ECMO after 3 days after showing improvement in oxygenation and ventilator mechanics.

Further workup was pursued, given the patient had sustained two prior episodes of ARDS and DAH in the past. Investigation revealed positive beta-2 glycoprotein (B2G) and lupus anticoagulant (LAC). Repeat imaging showed left cerebellar stroke, peripheral hypodensities in the spleen, kidneys, and liver, compatible with infarctions, and nonocclusive thrombus within the right external iliac vein. Rheumatology consultation was obtained, and the patient was diagnosed with catastrophic antiphospholipid syndrome (CAPS). Pulse-dose steroids were continued as well as intravenous immunoglobulin. The patient was later discharged without neurological sequelae with a tapering dose of steroids, and he was started on warfarin indefinitely.

## 3. Discussion

Prone positioning due to respiratory failure and ARDS poses challenges when patients sustain cardiac arrest [1, 2]. Considering the significant effort and time required to reposition patients from prone to supine during cardiac arrest, CPR may be required while patients are in prone position. Per the American Heart Association (AHA), prone position CPR is a reasonable measure for mechanical ventilated (with an advanced airway) patients who are unable to be safely returned to a supine position [3]. There are limited data and guidelines to standardize CPR performance while patients are in a prone position. It is recommended that patients should be placed on hard surfaces and compressions should be performed in the midline of thoracic spine between T7 and T9, using the same technique as supine CPR [4]. Though standard CPR while in supine position is ideal, Wei et al. concluded that prone CPR can generate higher mean blood pressure when compared to other measures such external cardiac massage [5]. In a small case series of six patients who underwent traditional CPR without return of ROSC, reverse CPR was subsequently done which showed an improvement in mean systolic blood pressure (32 mmHg standard CPR vs. 46 mmHg reverse CPR) and mean arterial blood pressure [6]. This observation however was limited given that neither group achieved ROSC in either positions. A large limitation to prone CPR is the inability to assess for adequate CPR quality and pulse between CPR rounds. Despite minimal reports regarding improvement in blood pressure with prone CPR, many intensivists may consider prioritizing adequate cardiovascular perfusion first followed by a potential attempt to place patients back to a supine position.

Bedside POCUS is a beneficial tool often used in critical care settings, especially during cardiac arrest. POCUS provides the ability to rapidly assess for reversible causes of cardiac arrest, guide interventions (intravenous access, pericardiocentesis), and provide monitoring for CPR quality [7]. Discrepancies in assessing pulse with manual palpation can delay necessary CPR or lead to inappropriate chest compressions when a pulse is present, both potentially increasing morbidity and mortality [8]. With variability during manual palpation of the carotid or femoral artery, compression of large arteries and assessment of pulsation with an ultrasound probe may provide more accurate information. Additionally, there is continued disagreement of the definition of cardiac standstill; however, pulsations in the carotid or femoral artery can likely indicate adequate cardiac contractility to generate peripheral pulses [8]. In a survey of 127 trainees and attending physicians across emergency medicine, critical care, and cardiology, significant variability was noted during interpretation of cardiac standstill during cardiac arrest [9]. If POCUS is used during ACLS, either the carotid or femoral arteries can be used in evaluating CPR quality during resuscitation [10]. In a small prospective study, POCUS with carotid artery compression was noted to significantly shorten ROSC determination when compared to manual palpation [10]. When assessing for central artery pulsation, both large veins and arteries will show absent pulses and will both be collapsible given the cardiovascular system is under a “no-flow state” [8]. To our current understanding, this is the first reported case in which POCUS has played a pivotal role in assisting in ACLS protocol during prone position compressions. Though not utilized within our case, implementation of pulse wave (PW) or color Doppler on ultrasound may provide beneficial data during CPR. In a small prospective pilot study, Koch et al. noted near physiological levels of peal systolic velocity within the carotid artery during CPR by utilizing PW [11]. Additionally noting that systolic forward flow and diastolic reversal by color Doppler within the carotid may provide a useful tool to assess the quality of CPR [11]. Using these tools with POCUS during CPR may provide better assessment of CPR, in addition to basic arterial compressibility and pulsatility.

Within our case, we utilized the linear-phase ultrasound probe for direct visualization of lower-extremity blood vessel (popliteal artery) contractility to successfully determine adequate prone CPR quality. Rapid ultrasound assessment with traditional strategies was not possible due to prone positioning in the setting of ARDS secondary to CAPS. Despite witnessing cardiac arrest, we determined limited time during no/low flow states even in the prone position because POCUS showed pulsations of the popliteal artery during chest compressions. Due to limited data on cardiac arrest due to respiratory insufficiency and post-ROSC ECMO outcomes, many ECMO teams/centers may not be comfortable in consistently cannulating patients for VV ECMO post-cardiac arrest due to the risk of patients developing anoxic brain injury and poor neurological outcomes. However, limited studies suggest that cardiac arrest prior to VV ECMO cannulation is not an independent predictor of death before hospital discharge [12, 13]. For patients who suffer cardiac arrest due to ARDS and refractory hypoxemia, Harnisch and Moerer suggest to look at the timing and adequacy of resuscitation rather than the resuscitation in itself [14]. Our POCUS findings of adequate perfusion during CPR and the likelihood of maintaining cerebral and multiorgan perfusion gave useful information to our ECMO team which ultimately played a part in early VV ECMO cannulation. Given the invasive nature of ECMO cannulation with potential high-risk complications, it is imperative to quickly determine candidacy for ECMO following cardiac arrest. POCUS during CPR can allow this rapid determination and decrease the delayed time of initiation to ECMO, further decreasing the risk for recurrent circulatory arrest. Overall, POCUS during CPR gave us the ability to adequately diagnose, manage, and treat our patient leading to his survival.

## 4. Conclusion

This case highlights the importance and feasibility of using POCUS to assess compression quality during prone position CPR, particularly by observing the popliteal artery pulsations. The ability to monitor perfusion through this method offers a valuable tool in guiding resuscitative efforts and potentially improving outcomes in patients sustaining cardiac arrest in prone positioning. Further studies and clinical application of POCUS in this context are warranted to validate its utility and integrate it into standard protocols for CPR. This innovative approach underscores the significance of adapting resuscitative techniques to unique clinical scenarios, aiming to optimize patient care and survival in critical situations.

## Data Availability

No data is included.
